# Children concurrently wasted and stunted: A meta‐analysis of prevalence data of children 6–59 months from 84 countries

**DOI:** 10.1111/mcn.12516

**Published:** 2017-09-25

**Authors:** Tanya Khara, Martha Mwangome, Moses Ngari, Carmel Dolan

**Affiliations:** ^1^ ENN Oxford UK; ^2^ KEMRI‐Wellcome Trust Research Programme KEMRI Centre for Geographic Medicine Research‐Coast Kilifi Kenya

**Keywords:** burden, children, concurrent wasting and stunting, global, prevalence

## Abstract

Children can be stunted and wasted at the same time. Having both deficits greatly elevates risk of mortality. The analysis aimed to estimate the prevalence and burden of children aged 6–59 months concurrently wasted and stunted. Data from demographic and health survey and Multi‐indicator Cluster Surveys datasets from 84 countries were analysed. Overall prevalence for being wasted, stunted, and concurrently wasted and stunted among children 6 to 59 months was calculated. A pooled prevalence of concurrence was estimated and reported by gender, age, United Nations regions, and contextual categories. Burden was calculated using population figures from the global joint estimates database. The pooled prevalence of concurrence in the 84 countries was 3.0%, 95% CI [2.97, 3.06], ranging from 0% to 8.0%. Nine countries reported a concurrence prevalence greater than 5%. The estimated burden was 5,963,940 children. Prevalence of concurrence was highest in the 12‐ to 24‐month age group 4.2%, 95% CI [4.1, 4.3], and was significantly higher among boys 3.54%, 95% CI [3.47, 3.61], compared to girls; 2.46%, 95% CI [2.41, 2.52]. Fragile and conflict‐affected states reported significantly higher concurrence 3.6%, 95% CI [3.5, 3.6], than those defined as stable 2.24%, 95% CI [2.18, 2.30]. This analysis represents the first multiple country estimation of the prevalence and burden of children concurrently wasted and stunted. Given the high risk of mortality associated with concurrence, the findings indicate a need to report on this condition as well as investigate whether these children are being reached through existing programmes.

## 1 INTRODUCTION

Reducing the prevalence of children under 5 years of age who are wasted and stunted is a global priority. Globally, an estimated 50 million children are wasted (16 million severely wasted), and 156 million are stunted (UNICEF, WHO, & Group, W. B., 2016). Each year, approximately 800,000 deaths are attributed to wasting (60% of which are attributable to severe wasting) and over 1 million to stunting. Wasting and stunting are also associated with the loss of 64.6 and 54.9 million disability adjusted life years, respectively, accounting for 14.8% and 12.6% of the total global disability adjusted life years for children under five (Black et al., 2008). Recent global analysis indicates that substantial progress in reducing the number of stunted children is being achieved but not in Africa (IFPRI, 2016). Globally, there has been less progress in reducing the number of wasted children (IFPRI, 2016). Overall, the world is off course to meet the World Health Assembly goals of a 40% reduction in the prevalence of stunted children and to reduce and maintain wasting at <5% by 2025 (IFPRI, 2016).

Recent reviews (Bergeron & Castleman, 2012; Briend, Khara, & Dolan, 2015; Khara & Dolan, 2014; Menon & Stoltzfus, 2012; Shoham, Dolan, & Gostelow, 2013) have highlighted the challenges associated with addressing stunting and wasting as separate issues, as has historically been the case. Current funding mechanisms can neglect the scale up of treatment of wasting and its prevention in long‐term developmental settings and, in emergency settings (especially those of a protracted nature), limit the attention to preventing increases in stunting. Research also misses opportunities to measure the impact of nutrition‐specific and nutrition‐sensitive interventions on both manifestations of undernutrition. Finally, programmes that focus separately on either wasting or stunting in the same context can lead to competition for resources and miss opportunities to optimise efforts to tackle both forms of undernutrition jointly.

Wasting and stunting are often present in the same geographical populations (Victora, 1992), and evidence suggests that they share many of the same underlying and basic causal factors (Martorell & Young, 2012). Research investigating whether there is a direct causal relationship between wasting and stunting is inconclusive, and a number of gaps in the evidence base have been highlighted (Angood, Khara, Dolan, Berkley, & WaSt TIG., 2016). It is recognised however that children can be stunted and wasted at the same time (Angood et al., 2016), and though the factors leading to this state of “concurrence” are poorly understood, evidence suggests that children with both deficits are at a greatly elevated risk of mortality (McDonald et al., 2013).

There are no global estimates of the prevalence and burden of concurrence (UNICEF et al., 2016), and it is rarely reported, though the data required to do so is readily available in national surveys (Saaka & Galaa, 2016). By reporting global figures for the prevalence of different nutritional deficits separately, first, the true proportion of the global population affected by nutritional deficits as a whole is underestimated, and second, the proportion of children under five affected by multiple deficits is missed.

These limitations have recently been recognised in the international literature (IFPRI, 2015, 2016; UNICEF et al., 2016). To address this knowledge gap, the authors conducted a preliminary analysis for the Global Nutrition Report 2015 using Demographic Health Survey (DHS) data from five countries. This analysis indicated that the prevalence of concurrence in these countries ranged from 2.9% to 7.4% and that less than 50% of children in those countries avoided either wasting or stunting (IFPRI, 2015). This paper builds on that analysis (IFPRI, 2016) by utilising population anthropometric data from 84 countries to (a) estimate the prevalence and burden of concurrence in countries with available data (aiming to approach a global estimate), (b) explore age, sex, regional, and contextual differences, and (c) give an estimate of the proportion of children affected by either of these conditions (wasted or stunted).

**Key messages**

- Children concurrently wasted and stunted experience a mortality risk similar to that of severely wasted children. However, the prevalence and burden of this condition is not systematically reported on nationally or internationally. We highlight the need to do so.
- This first analysis of the prevalence of children (6–59 months) concurrently wasted and stunted in 84 countries indicates that 9 countries have levels of >5%. These levels are concerning given the mortality risk.
- Our 84 country pooled estimate of 3% corresponds to nearly 6 million children, underlining the need to investigate whether these children are being reached appropriately through existing interventions.

## 2 ETHICS STATEMENT AND METHODS

Approval to access the datasets was obtained through online registration to DHS Macro (http://dhsprogram.com/data/Access-Instructions.cfm) and from United Nations Children's Fund (UNICEF) for Multi‐indicator Cluster Surveys (MICS). DHS and MICS surveys are a well‐established and respected global initiatives conducted with appropriate in‐country permissions and informed consents. Ethical approval for the analyses presented here was not sought as the paper is based on anonymous data provided for the purposes of secondary analysis research.

### 2.1 Data sources

Two sources of nationally representative population level data were identified: (a) the DHS data http://dhsprogram.com/data/ and (b) the MICS data http://mics.unicef.org/surveys. Standard DHS are usually large (5,000 to 30,000 households) nationally representative surveys conducted approximately every 5 years. The DHS covers a wide range of topics, but for this study, the variables of interest were from the child nutrition survey topic. Both DHS and MICs datasets were included because they have the anthropometric variables of interest, have standard methodologies, are considered nationally representative, and are systematically carried out. Both sources also form a large part of the UNICEF/World Health Organization (WHO) joint estimates database in which they are combined as part of the estimation of global burden figures for malnutrition (UNICEF et al., 2016).

We included data of children aged 6–59 months collected between 2005 and 2015 (10‐year period). For countries with more than one dataset available between the time references, the most recent dataset was chosen. Data were downloaded and imported into STATA Version 13.0 for analysis.

### 2.2 Measures

The primary measures were children stunted defined as those with height‐for‐age *z*‐score (HAZ) < −2, children wasted defined as those with weight‐for‐height/length *z*‐score (WHZ) < −2, and children concurrently wasted and stunted defined as HAZ < −2 and WHZ < −2, respectively. The *z*‐scores were computed using the WHO 2016 growth reference standards using the STATA macro (WHO, 2006). Children were considered to be free from either condition if their WHZ > −2 and HAZ > −2.

Five age categories were defined: less than 12 months, 12 to 24 months, 24 to 36 months, 36 to 48 months, and 48 to 60 months.

The five United Nations geographical regions and subregions were used to define geographic regions (http://unstats.un.org/unsd/methods/m49/m49regin.htm), and the fragile and conflict‐affected states (FCAS) country classification was adopted from the UK Department for International Development (DFID, 2015).

### 2.3 Data cleaning

A two‐stage approach to anthropometric data cleaning was applied: (a) biological plausibility criteria where values were set to missing if weight > 50 kg or if height > 200 cm and (b) WHO statistical probability criteria (Crowe, Seal, Grijalva‐Eternod, & Kerac, 2014) where HAZ was set to missing if HAZ > 6 or < −6 and WHZ was set to missing if WHZ > 5 or < −5. Additionally, any record with missing parameters for the calculation of WHZ or HAZ was dropped from the analysis (Crowe et al., 2014).

### 2.4 Data analysis

#### 2.4.1 Prevalence of wasting, stunting, and concurrence

The overall prevalence and binomial exact 95% confidence intervals (CI) for being wasted, stunted, concurrently wasted and stunted, and wasted or stunted were calculated. Country‐specific estimates were calculated and pooled using the random‐effects meta‐analysis to yield the 84 country estimates (Hamza, Reitsma, & Stijnen, 2008). Even though the distribution of countries specific wasting–stunting prevalence was not expected to be normal, random effect meta‐analysis pooled estimate would not be biased because of the study large sample (*N* = 570,930), and this approach has previously been used to pool large samples estimate of DHS data from many countries (Akombi, Agho, Merom, Renzaho, & Hall, 2017; Neupane, Prakash, & Doku, 2016; Wamani, Astrøm, Peterson, Tumwine, & Tylleskär, 2007). A sensitivity analysis was performed by cumulating all the countries data and calculated the pooled estimate as number of children with concurrent wasting and stunting divided by the total *N* (*N* = 570,930) and compared with the random effect estimate. Country‐specific prevalence was also pooled by gender, age, United Nations regions, FCAS classification, and WHO population classification of severity of wasting and stunting. Heterogeneity chi‐squared values and inconsistency (*I* ^2^) statistic were used to quantify the countries prevalence heterogeneity. Test of proportion was used to test differences in the prevalence within groups using the 84 country pooled prevalence as the reference.

#### 2.4.2 Burden of concurrent wasting and stunting

To calculate the burden of concurrent wasting and stunting, we took the 0‐ to 59‐month‐old country population from the UNICEF/WHO/World Bank joint estimates database, updated in 2015 (UNICEF et al., 2016). We assumed that infants under 6 months were one tenth of the total 0‐ to under 60‐month population (Division, 2015) therefore multiplied the country population figures by 90% to arrive at the 6‐ to 59‐month‐old population estimate. The 84 country burden of concurrence was then estimated by multiplying the pooled 84 country prevalence by the total population estimate for 6‐ to 59‐month‐old children within the 84 countries.

## 3 RESULTS

In total, 84 countries had datasets eligible for analysis. These countries were from 5 United Nations regions; 40 datasets were from Africa, 25 from Asia, 12 from Latin America, 6 from Europe, and 1 from Oceania. After selecting the most recent dataset available for each country, we arrived at 46 DHS datasets and 38 MICS datasets. A total number of 570,930 children aged between 6 and 59 months were included into the analysis; 290,073 (51%) were males.

### 3.1 Prevalence and burden of concurrence

The pooled prevalence of children concurrently wasted and stunted in the 84 countries was 3.0%, 95% CI [2.97, 3.06] (Table 1). The pooled wasting–stunting estimate from the sensitivity analysis was similar (prevalence 3.0%, 95% CI [2.97, 3.06]). The prevalence of concurrence varied across countries from 0% in Montenegro to 8.0%, 95% CI [7.2, 8.9], in Niger (*I* ^2^ = 99.8% and *p* value < .0001). Nine countries had a concurrence prevalence greater than 5%. Six from sub‐Saharan Africa (Niger, Burundi, Djibouti, Chad, Sudan, and South Sudan) and three from Asia (Timor‐Leste, Yemen, and India; Figure S1).

**Table 1:** Pooled prevalence of anthropometric deficits in children (6–59 months) in 84 countries

Categories of anthropometric deficit	Prevalence	Lower CI	Upper CI
Not wasted, not stunted	61.1	61.0	61.3
Wasted	8.8	8.7	8.9
Stunted	33.0	32.9	33.2
Concurrently wasted and stunted	3.0	3.0	3.1
Wasted or stunted	38.9	38.7	39.0

*Note*. CI = confidence interval.

The total population of children aged 6 to 59 months among the 84 countries included in this analysis was 198,005,973. The burden of concurrence among children aged 6 to 59 months was estimated to be 5,963,940 children (Table S1).

**Figure 1:**
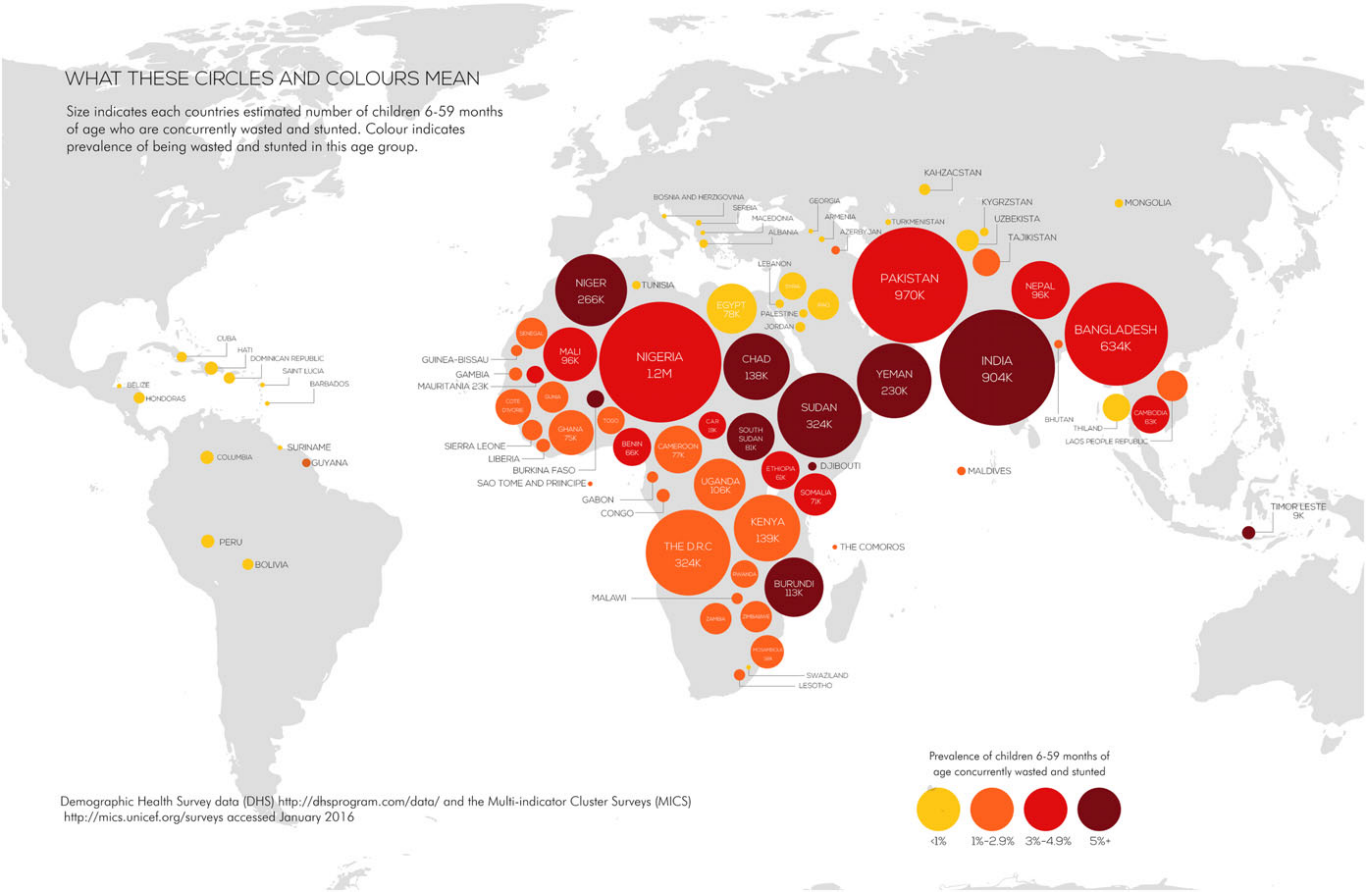
Prevalence and burden of children 6 to 59 months of age concurrently wasted and stunted in 84 countries. [Correction added on 05 October 2017, after first online publication: An incorrect version of Figure 1 was used and has now been corrected. Figure caption updated accordingly]

The country prevalence and burden of concurrence for all 84 countries is illustrated in Figure 1.

### 3.2 Prevalence of concurrence by age and gender

The prevalence of children concurrently wasted and stunted was observed to be significantly higher in the 12‐ to 24‐month age group, 4.2%, 95% CI [4.1 to 4.3], and the 24‐ to 36‐month age group, 3.2%, 95% CI [3., 3.3] (test of proportions *p* value < .001; Table 2 **.**). Gender differences were also observed with a significantly higher prevalence of concurrence among males, 3.54%, 95% CI [3.47, 3.61], compared to females, 2.46%, 95% CI [2.41, 2.52] (test of proportion *p* value < .001).

**Table 2:** Age distribution of concurrent wasting and stunting in 84 countries

Age group (months)	Total (*N*)	%	No. of cases of concurrence (*n*)	Prevalence of concurrence(*n / N*) * 100	LCI	UCI	*p* value
6–12	65,495	11.47	1,562	2.39	2.26	2.51	.001
12–24	127,831	22.39	5,394	4.22	4.12	4.33	.001
24–36	127,012	22.25	4,108	3.23	3.14	3.33	.001
36–48	129,368	22.66	3,257	2.52	2.43	2.61	.001
48–60	121,224	21.23	2,875	2.37	2.28	2.46	.001
Total	570,930	100	17,196				

*Note*. LCI = lower confidence interval; UCI = upper confidence interval; *p* value = test of within group heterogeneity.

When wasting and stunting were analysed separately for girls and boys, we also found higher prevalence among males, wasted prevalence for boys, 9.5%, 95% CI [9.3, 9.6], and girls, 8.1%, 95% CI [8.0, 8.2], *p* value < .001; stunted prevalence for boys, 34.3% 95% CI [34.1, 34.5], compared to girls, 31.7%, 95% CI [31.5, 31.8], *p* value < .001.

### 3.3 Prevalence of concurrence by United Nations classification of regions

**Figure 2:**
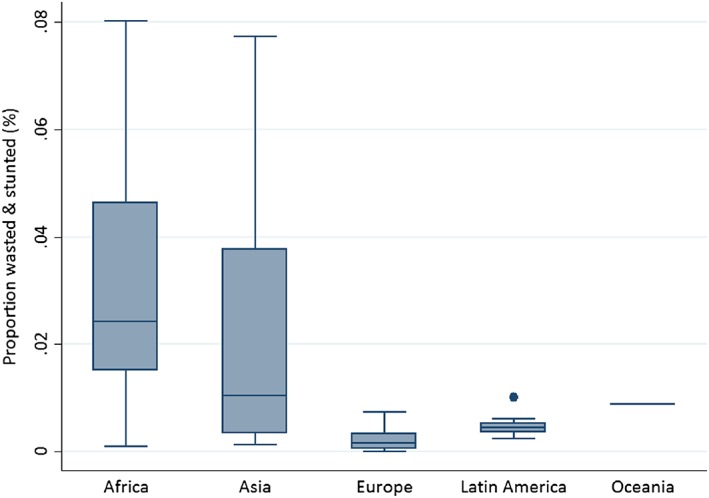
Box plot of proportion (maximum and minimum) of concurrence within the United Nations regions

The prevalence of concurrence was highest in the Africa region, 3.5%, 95% CI [3.4, 3.6], and Asia regions, 3.4%, 95% CI [3.3, 3.5]. Within Africa, the West Africa subregion registered the highest prevalence, 3.9%, 95% CI [3.8, 4.1], and within Asia, South Asia registered the highest prevalence, 4.4%, 95% CI [4.3, 4.5] (Figure 2, Table S2).

### 3.4 Prevalence of concurrence in FCAS

Of the 84 countries included in this analysis, 41 were classified as FCAS. The pooled prevalence of concurrence in these FCAS was 3.6%, 95% CI [3.5, 3.6], significantly higher than the estimate in contexts defined as stable, which was 2.24%, 95% CI [2.18, 2.30%] (test of proportion *p* value < .0001).

### 3.5 Prevalence of concurrence by WHO categories of severity of wasting and stunting

We used the WHO definition of severity of wasting and stunting at population level (WHO, 2010) to categorise the 84 countries in our sample. By this criteria, 7/84 countries (8.3%) had levels of wasting classified as “serious” or “critical,” and 34/84 countries (40%) had levels of stunting classified as “high” or “very high.” As might be expected, the prevalence of concurrence was significantly higher; 5.2%, 95% CI [5.0, 5.4], and 5.8%, 95% CI [5.3, 6.4] in the countries that had serious (10–15%) and critical (>15%) levels of wasting, respectively. Similarly, the pooled prevalence of concurrence was significantly higher in the 34 countries with high (30–40%) and very high (>40%) prevalence of stunting (Table 3.)

**Table 3:** Concurrence by public health severity of wasting and stunting

Categories of wasting	Definition	Number of countries	Pooled prevalence of concurrent wasting and stunting (95% CI)	*p* value
<5% wasting	Acceptable	49	1.084 (1.045 to 1.123)	<.001
5–9.99%	Alarming	28	4.849 (4.760 to 4.938)	<.001
10–14.99%	Serious	5	5.204 (5.011 to 5.396)	<.001
>15%	Critical	2	5.872 (5.313 to 6.432)	<.011
Categories of stunting				
<20%	Low	25	0.543 (0.501 to 0.585)	<.001
20–29.99%	Medium	25	1.817 (1.743 to 1.891)	<.001
30–39.99%	High	22	4.901 (4.809 to 4.992)	<.001
>40%	Very high	12	3.575 (3.459 to 3.690)	<.001

*Note*. CI = confidence interval; *p* value = Test of heterogeneity among countries with the groups.

### 3.6 Prevalence of children wasted or stunted

The pooled 84 country prevalence estimate for children 6–59 months of age experiencing either wasting or stunting was 38.9%, 95% CI [38.7, 39.0]. This means that only 61.1%, 95% CI [61.0, 61.3], of children in the 84 countries escape both conditions (Table 1.).

## 4 DISCUSSION

Previous research indicates the heightened risk of mortality for children with multiple anthropometric deficits (McDonald et al., 2013). A child who is both wasted and stunted is 12 times more likely to die than a child who is neither wasted nor stunted. This is a similarly high risk of mortality to that estimated for severe wasting (Olofin et al., 2013), a condition prioritised for routine identification and therapeutic treatment according to international protocols (WHO, WFP, UNSCN, & UNICEF, 2007). In this analysis, we set out to estimate the 84 country prevalence and burden of concurrence among children aged 6 to 59 months and their age, gender, and regional variation.

Although the pooled prevalence of children with concurrence was 3.0%, we found that there is considerable variation between countries. Nine countries have prevalence rates of over 5%. A country prevalence of >5% severe wasting would warrant concern and intensification of efforts to identify and treat children. Though concurrence is associated with similar mortality risks, its prevalence is not monitored, and cases are not routinely identified, therefore, no specific action can be taken.

Apart from the previous five country analysis carried out by the authors (IFPRI, 2015), there is little published data on concurrence for comparison with our analysis. In a recent reported analysis of 2014 DHS data from Ghana, a lower prevalence of 1.4% was reported than the 2.2% found in our analysis. However, infants 0–6 months old were included in the analysis (Saaka & Galaa, 2016).

Our analysis was not able to explore within country regional prevalence differences, but these may be considerable. In the Ghana analysis, significant within country geographical variation in concurrence was reported with a high of 3.2%, 95% CI [1.7–5.8], and a low of 0.5%, 95% CI [0.1–3.7] (Saaka & Galaa, 2016). This finding suggests that reported national averages can mask pockets of much poorer nutritional status within countries leading to possible underestimation of the burden of wasting and stunting within a country.

We have used cross‐sectional data to capture the intersection of wasting and stunting in this analysis. However, given the transitory nature of wasting in particular, where a child can experience several episodes of wasting during a set period, using cross‐sectional data insufficiently estimates the actual prevalence (Garenne et al., 2009). This means that we are likely to be underestimating the true burden of children experiencing these two deficits concurrently. Despite this, the levels reported in our analysis suggest that determining the extent to which children with concurrence are being reached and how this might be improved should be a priority for researchers, programmers, and policy makers.

We found that when pooled prevalence for concurrence are generated for specific contexts, countries classified as FCAS have a significantly higher prevalence compared to more stable countries. As a large proportion of children concurrently wasted and stunted reside in FCAS where their access to basic treatment or preventative services will be particularly constrained, this finding underlines the need to investigate the extent to which existing programming, either for the treatment of acute malnutrition or prevention of wasting or stunting, in these contexts, are reaching these children effectively.

Our findings that younger children (12–36 months of age) had a greater risk of concurrence is likely to be due to the typically higher prevalence of stunting and wasting individually found in these age groups. The relatively lower risk in the 6‐ to12‐month age group however contrasts with the Ghana data where a higher risk of stunting in wasted children was moderated by age with the strongest association in the 0‐ to 5‐month‐old and 12‐ to 23‐month‐old children (Saaka & Galaa, 2016).

We found that boys were significantly more likely to be concurrently wasted and stunted than girls. This mirrors significantly higher prevalence of wasting and of stunting in boys when analysed separately. That boys can be more nutritionally vulnerable than girls is commonly reported in nutrition surveys, but this is rarely considered when formulating nutrition policies and programmes. It is also not considered in nutrition related gender policies. The Scaling Up Nutrition Movement Road Map for 2016–2020, for example, makes explicit reference to “adopt policies that reduce nutritional inequities, especially among women and girls and eliminate discriminatory laws and practices” (Secretariat, 2016). The causes of this apparent heightened vulnerability among boys needs further investigation so that policy and programme adjustments, if warranted, can be made to support better linear and ponderal growth.

There is some indication both in the Ghana analysis (Saaka & Galaa, 2016) and the wider literature (Dewey et al., 2005; Doherty et al., 2001; Richard et al., 2012; Walker, Grantham‐McGregor, Himes, & Powell, 1996) that linear growth is affected during periods of wasting. The cross‐sectional nature of our data means that it is of limited value in exploring this. The question of whether wasting is a risk factor for stunting and vice versa requires further investigation using longitudinal data, which can reflect this age group's complete experience of these deficits.

Finally, our finding that over two thirds (38%) of children 6–59 months of age in the 84 countries are either stunted or wasted provides a very stark metric for understanding the extent of undernutrition in these countries. From country disaggregated data from our analysis reported in the Global Nutrition Report 2016, we can also see that in a number of countries (Benin, Djibouti, Yemen, Niger, Chad, Guinea‐Bissau, Ethiopia, Congo DRC, Burundi and Somalia, India, Pakistan, and Laos), over half the population of children is suffering from one of these deficits (IFPRI, 2016).

Country and global reporting systems need to report the combined extent of wasting and stunting in the 6‐ to 59‐month population in order to provide a more holistic picture of the proportion of children under 5 years of age affected by undernutrition to highlight their needs and monitor overall progress.

### 4.1 STUDY STRENGTH AND LIMITATIONS

Our study provides multicountry estimates for concurrence not previously reported either in the grey or peer reviewed literature. We were able to obtain nationally representative data from the last 10 years for 84 countries; therefore, we cannot assume our results approximate to global estimates. However, the spread of countries in our dataset from different regions and contexts suggests a global prevalence may be similar.

The limitations of the use of cross‐sectional data in estimating prevalence of wasting are noted in the discussion. In addition, the use of DHS and MICS surveys introduce a number of limitations. The data spans a 10‐year period; surveys are done as different times of year and weight, age, or height might be incorrectly measured or reported. These biases would affect intercountry comparisons and overall prevalence estimates.

## CONFLICTS OF INTEREST

The authors declare that they have no conflicts of interest.

## CONTRIBUTIONS

TK, MM, and CD conceptualised the study. TK drafted the initial manuscript. MM and MN performed the main data analysis. All authors contributed to the development of the final manuscript.

## Supporting information


**Supplemental Figure 1**: Country prevalence's (95% CI) of children aged 6 to 59 months concurrently wasted and stunted

Supplemental Table 1. Countries contribution to the estimated burden of concurrent wasting and stunting among children 6 to 59 months' old
Supplemental Table 2. Regional and sub‐regional distribution of concurrent wasting and stunting
