# Supplementary figures and images for: Children concurrently wasted and stunted: A meta‐analysis of prevalence data of children 6–59 months from 84 countries
Source: Matern Child Nutr. 2017 Sep 25;14(2):e12516. doi: 10.1111/mcn.12516 (PMC5901398; doi:10.1111/mcn.12516)

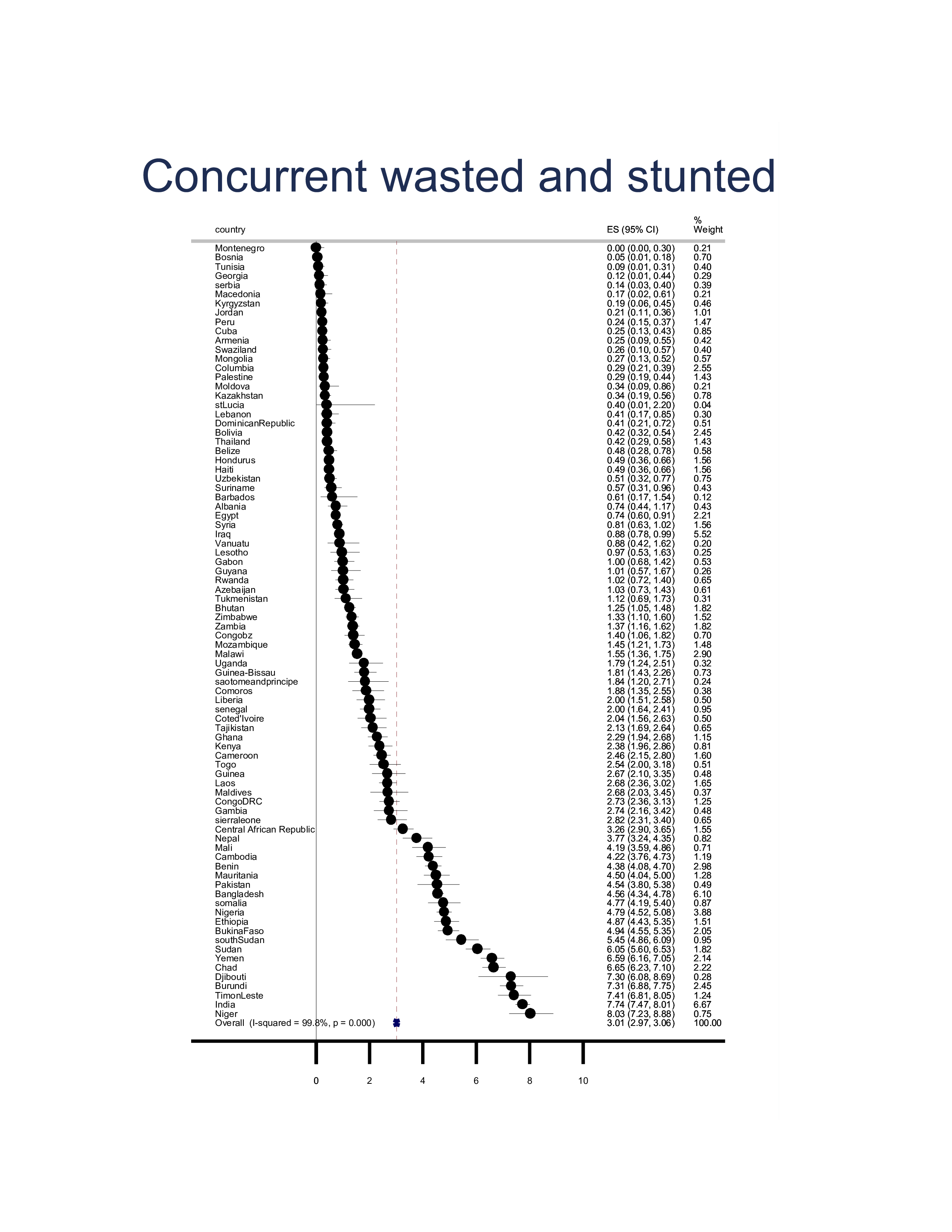

Supplement: Supplementary file 1 — Supplemental Figure 1: Country prevalence's (95% CI) of children aged 6 to 59 months concurrently wasted and stunted [file MCN-14-e12516-s001.tiff]
