# Supplementary material for: Children concurrently wasted and stunted: A meta‐analysis of prevalence data of children 6–59 months from 84 countries
Source: Matern Child Nutr. 2017 Sep 25;14(2):e12516. doi: 10.1111/mcn.12516 (PMC5901398; doi:10.1111/mcn.12516)
Supplement: Supplementary file 2 — Supplemental Table 1. Countries contribution to the estimated burden of concurrent wasting and stunting among children 6 to 59 months' old Supplemental Table 2. Regional and sub‐regional distribution of concurrent wasting and stunting [file MCN-14-e12516-s002.docx]

|  | Country | Prevalence | N (0 to 59 months) | Burden (0 to 59 months) | N (6 to 59 months) | Burden  (6-59 months) |
| --- | --- | --- | --- | --- | --- | --- |
| 1 | NIGERIA | 0.0452 | 29816179 | 1347691 | 26834561.1 | 1212922 |
| 2 | PAKISTAN | 0.0454 | 23740714 | 1077828 | 21366642.6 | 970046 |
| 3 | INDIA | 0.0774 | 12983535 | 1004926 | 11685181.7 | 904433 |
| 4 | BANGLADESH | 0.0456 | 15448776 | 704464 | 13903898.4 | 634018 |
| 5 | DEMOCRATIC REP. OF THE CONGO (THE) | 0.0273 | 13222905 | 360985 | 11900614.5 | 324887 |
| 6 | SUDAN (THE) | 0.0605 | 5655381 | 342151 | 5089842.9 | 307936 |
| 7 | NIGER (THE) | 0.0803 | 3686758 | 296047 | 3318082.2 | 266442 |
| 8 | YEMEN | 0.0659 | 3885141 | 256031 | 3496626.9 | 230428 |
| 9 | KENYA | 0.0238 | 6512888 | 155007 | 5861599.2 | 139506 |
| 10 | CHAD | 0.0665 | 2313952 | 153878 | 2082556.8 | 138490 |
| 11 | BURUNDI | 0.0731 | 1722486 | 125914 | 1550237.4 | 113322 |
| 12 | UGANDA | 0.0179 | 6614726 | 118404 | 5953253.4 | 106563 |
| 13 | NEPAL | 0.0377 | 2854206 | 107604 | 2568785.4 | 96843 |
| 14 | MALI | 0.0419 | 2553458 | 106990 | 2298112.2 | 96291 |
| 15 | SOUTH SUDAN | 0.0545 | 1655516 | 90226 | 1489964.4 | 81203 |
| 16 | EGYPT | 0.0074 | 11727248 | 86782 | 10554523.2 | 78103 |
| 17 | CAMEROON | 0.0246 | 3503784 | 86193 | 3153405.6 | 77574 |
| 18 | GHANA | 0.0229 | 3672362 | 84097 | 3305125.8 | 75687 |
| 19 | SOMALIA | 0.0477 | 1654703 | 78929 | 1489232.7 | 71036 |
| 20 | BENIN | 0.0438 | 1681018 | 73629 | 1512916.2 | 66266 |
| 21 | CAMBODIA | 0.0422 | 1678126 | 70817 | 1510313.4 | 63735 |
| 22 | COTE D'IVOIRE | 0.0204 | 3426228 | 69895 | 3083605.2 | 62906 |
| 23 | ETHIOPIA | 0.0487 | 1401047 | 68231 | 1260942.3 | 61408 |
| 24 | MOZAMBIQUE | 0.0145 | 4444951 | 64452 | 4000455.9 | 58007 |
| 25 | GUINEA | 0.0267 | 1918815 | 51232 | 1726933.5 | 46109 |
| 26 | SENEGAL | 0.0199 | 2529321 | 50334 | 2276388.9 | 45300 |
| 27 | IRAQ | 0.00879 | 5139806 | 45179 | 4625825.4 | 40661 |
| 28 | ZAMBIA | 0.0137 | 2732145 | 37430 | 2458930.5 | 33687 |
| 29 | ZIMBABWE | 0.0133 | 2460013 | 32718 | 2214011.7 | 29446 |
| 30 | TOGO | 0.0254 | 1142489 | 29019 | 1028240.1 | 26117 |
| 31 | SIERRA LEONE | 0.0282 | 993297 | 28011 | 893967.3 | 25210 |
| 32 | MAURITANIA | 0.045 | 574736 | 25863 | 517262.4 | 23277 |
| 33 | LAO PEOPLE'S DEMOCRATIC REP. (THE) | 0.0268 | 830683 | 22262 | 747614.7 | 20036 |
| 34 | CENTRAL AFRICAN REPUBLIC (THE) | 0.0326 | 656253 | 21394 | 590627.7 | 19255 |
| 35 | SYRIAN ARAB REPUBLIC (THE) | 0.00808 | 2421389 | 19565 | 2179250.1 | 17608 |
| 36 | TAJIKISTAN | 0.0213 | 861055 | 18341 | 774949.5 | 16506 |
| 37 | THAILAND | 0.00417 | 4345764 | 18122 | 3911187.6 | 16310 |
| 38 | RWANDA | 0.0102 | 1682697 | 17164 | 1514427.3 | 15447 |
| 39 | BURKINA FASO | 0.0494 | 285699 | 14114 | 257129.1 | 12702 |
| 40 | LIBERIA | 0.0199 | 685659 | 13645 | 617093.1 | 12280 |
| 41 | UZBEKISTAN | 0.00512 | 2564055 | 13128 | 2307649.5 | 11815 |
| 42 | COLOMBIA | 0.0029 | 3929005 | 11394 | 3536104.5 | 10255 |
| 43 | TIMOR-LESTE | 0.074 | 149169 | 11039 | 134252.1 | 9935 |
| 44 | CONGO (THE) | 0.01402 | 695086 | 9745 | 625577.4 | 8771 |
| 45 | GAMBIA (THE) | 0.0273 | 346272 | 9453 | 311644.8 | 8508 |
| 46 | PERU | 0.002386 | 2972781 | 7093 | 2675502.9 | 6384 |
| 47 | HAITI | 0.00494 | 1253139 | 6191 | 1127825.1 | 5572 |
| 48 | KAZAKHSTAN | 0.003389 | 1602525 | 5431 | 1442272.5 | 4888 |
| 49 | BOLIVIA (PLURINATIONAL STATE OF) | 0.004154 | 1188482 | 4937 | 1069633.8 | 4443 |
| 50 | MALAWI | 0.015469 | 289527 | 4479 | 260574.3 | 4031 |
| 51 | DOMINICAN REPUBLIC (THE) | 0.00413 | 1063559 | 4393 | 957203.1 | 3953 |
| 52 | GUINEA-BISSAU | 0.018094 | 242502 | 4388 | 218251.8 | 3949 |
| 53 | HONDURAS | 0.00494 | 848597 | 4192 | 763737.3 | 3773 |
| 54 | LESOTHO | 0.00973 | 251643 | 2449 | 226478.7 | 2204 |
| 55 | GABON | 0.01 | 226668 | 2267 | 204001.2 | 2040 |
| 56 | JORDAN | 0.002086 | 926724 | 1933 | 834051.6 | 1740 |
| 57 | CUBA | 0.00246 | 749264 | 1843 | 674337.6 | 1659 |
| 58 | LEBANON | 0.00411 | 328826 | 1352 | 295943.4 | 1216 |
| 59 | KYRGYZSTAN | 0.00192 | 691795 | 1328 | 622615.5 | 1195 |
| 60 | ALBANIA | 0.0074 | 163573 | 1210 | 147215.7 | 1089 |
| 61 | BHUTAN | 0.0125 | 71967 | 900 | 64770.3 | 810 |
| 62 | MALDIVES | 0.0267 | 31964 | 853 | 28767.6 | 768 |
| 63 | TUNISIA | 0.00087 | 914799 | 796 | 823319.1 | 716 |
| 64 | MONGOLIA | 0.002745 | 280323 | 770 | 252290.7 | 693 |
| 65 | DJIBOUTI | 0.073 | 10066 | 735 | 9059.4 | 661 |
| 66 | GUYANA | 0.0101 | 67784 | 685 | 61005.6 | 616 |
| 67 | AZERBAIJAN | 0.01034 | 62253 | 644 | 56027.7 | 579 |
| 68 | SERBIA | 0.001359 | 453367 | 616 | 408030.3 | 555 |
| 69 | TURKMENISTAN | 0.00112 | 489938 | 549 | 440944.2 | 494 |
| 70 | ARMENIA | 0.00251 | 215886 | 542 | 194297.4 | 488 |
| 71 | SAO TOME AND PRINCIPE | 0.0184 | 27084 | 498 | 24375.6 | 449 |
| 72 | SWAZILAND | 0.0026 | 164116 | 427 | 147704.4 | 384 |
| 73 | SURINAME | 0.00575 | 49195 | 283 | 44275.5 | 255 |
| 74 | GEORGIA | 0.0012 | 234864 | 282 | 211377.6 | 254 |
| 75 | VANUATU | 0.0088 | 30912 | 272 | 27820.8 | 245 |
| 76 | BELIZE | 0.0048 | 37531 | 180 | 33777.9 | 162 |
| 77 | BARBADOS | 0.00606 | 17316 | 105 | 15584.4 | 94 |
| 78 | BOSNIA AND HERZEGOVINA | 0.0005 | 189273 | 95 | 170345.7 | 85 |
| 79 | SAINT LUCIA | 0.00398 | 14089 | 56 | 12680.1 | 51 |
| 80 | COMOROS (THE) | 0.0188 | 1134 | 21 | 1020.6 | 19 |
| 81 | MONTENEGRO | 0 | 38444 | 0 | 34599.6 | 0 |
| 82 | MOLDOVA | 0.0033585 | 225730 | 758 | 203157.0 | 682 |
| 83 | MACEDONIA | 0.0016849 | 110625 | 186 | 99562.5 | 168 |
| 84 | PALESTINE | 0.0029465 | 668876 | 1971 | 601988.4 | 1774 |
|  | Totals |  | 220,006,637 | 6,626,600 | 198,005,973 | 6746423 |
|  | Pooled prevalence | 0.03012 |  |  |  |  |
|  | **Burden** |  |  | | 198005973*0.03012 = **5,963,940** | |

**Supplemental Table 1. Countries contribution to the estimated burden of concurrent wasting and stunting among children 6 to 59 months’ old**

| **UN regions and sub-regions (number of countries)** | **Prevalence** | **Lower CI** | **Upper CI** | **P value** |
| --- | --- | --- | --- | --- |
|  |  |  |  |  |
| ***Africa (40)*** | **3.509** | **3.439** | **3.580** | <0.0001 |
| East Africa (13) | 3.240 | 3.124 | 3.356 | <0.0001 |
| Middle Africa (7) | 3.601 | 3.429 | 3.773 | <0.0001 |
| West Africa (15) | 3.985 | 3.862 | 4.107 | <0.0001 |
| North Africa (3) | 2.864 | 2.658 | 3.069 | <0.0001 |
| South Africa (2) | 0.536 | 0.280 | 0.792 | 0.006 |
|  |  |  |  |  |
| ***Asia (25)*** | **3.412** | **3.336** | **3.488** | <0.0001 |
| Central Asia (6) | 0.701 | 0.592 | 0.809 | <0.0001 |
| South Asia (8) | 4.430 | 4.319 | 4.541 | <0.0001 |
| West Asia (8) | 2.182 | 1.046 | 2.318 | <0.0001 |
| South East (2) | 3.326 | 3.046 | 3.606 | <0.0001 |
| East Asia (1) | 0.274 | 0.077 | 0.472 | -* |
|  |  |  |  |  |
| ***Latin America (12)*** | **0.399** | **0.350** | **0.448** | 0.001 |
| Caribbean (5) | 0.415 | 0.313 | 0.516 | 0.191 |
| South America (5) | 0.365 | 0.304 | 0.427 | 0.004 |
| Central America (2) | 0.491 | 0.361 | 0.621 | 0.939 |
|  |  |  |  |  |
| ***Europe (6)*** | **0.238** | **0.139** | **0.336** | <0.0001 |
| Southern Europe (5) | 0.227 | 0.126 | 0.327 | <0.0001 |
| Eastern Europe (1) | 0.336 | -0.047 | 0.719 | -* |
|  |  |  |  |  |
| ***Ocenia(1)*** | **0.884** | **0.425** | **1.620** | -* |
| Melanesi (1) | 0.884 | 0.287 | 1.482 | -* |
|  |  |  |  |  |
| ¶-Test of within group heterogeneity P-value, *-Missing p-values, few numbers to generate P-values, | | | | |

**Supplemental Table 2. Regional and sub-regional distribution of concurrent wasting and stunting**
